# Intra-individual Gene Expression Variability of Histologically Normal Breast Tissue

**DOI:** 10.1038/s41598-018-27505-y

**Published:** 2018-06-14

**Authors:** Xuezheng Sun, Yue Shan, Quefeng Li, Lynn Chollet-Hinton, Erin L. Kirk, Gretchen L. Gierach, Melissa A. Troester

**Affiliations:** 1 Department of Epidemiology, Gillings School of Public Health, University of North Carolina, Chapel Hill, USA; 2 Center for Environmental Health and Susceptibility, University of North Carolina, Chapel Hill, USA; 3 Department of Biostatistics, Gillings School of Public Health, University of North Carolina, Chapel Hill, USA; 4 Lineberger Comprehensive Cancer Center, University of North Carolina, Chapel Hill, USA; 5 Metabolic Epidemiology Branch, Division of Cancer Epidemiology & Genetics, National Cancer Institute, Rockvill, USA

## Abstract

Several studies have sought to identify novel transcriptional biomarkers in normal breast or breast microenvironment to predict tumor risk and prognosis. However, systematic efforts to evaluate intra-individual variability of gene expression within normal breast have not been reported. This study analyzed the microarray gene expression data of 288 samples from 170 women in the Normal Breast Study (NBS), wherein multiple histologically normal breast samples were collected from different block regions and different sections at a given region. Intra-individual differences in global gene expression and selected gene expression signatures were quantified and evaluated in association with other patient-level factors. We found that intra-individual reliability was relatively high in global gene expression, but differed by signatures, with composition-related signatures (i.e., stroma) having higher intra-individual variability and tumorigenesis-related signatures (i.e., proliferation) having lower intra-individual variability. Histological stroma composition was the only factor significantly associated with heterogeneous breast tissue (defined as > median intra-individual variation; high nuclear density, odds ratio [OR] = 3.42, 95% confidence interval [CI] = 1.15–10.15; low area, OR = 0.29, 95% CI = 0.10–0.86). Other factors suggestively influencing the variability included age, BMI, and adipose nuclear density. Our results underscore the importance of considering intra-individual variability in tissue-based biomarker development, and have important implications for normal breast research.

## Introduction

Breast tissue changes over a woman’s lifetime, altered by endogenous and exogenous factors. Previous studies have evaluated gene expression and histological alterations in association with putative breast cancer risk factors, such as age, obesity, and mammographic density, and their findings provide important insights into breast cancer etiology^1–8^. In addition, recent research has characterized changes in histologic and molecular features of benign tissue adjacent to breast tumor, and linked these changes to breast cancer outcomes^9–12^, demonstrating the potential for benign breast tissue to inform breast cancer outcomes or risk stratification of pre-cancerous breast. For further development of biomarkers based on benign breast gene expression, careful consideration must be given to biomarker characteristics.

Low intra-individual and high inter-individual variabilities are important properties of reliable biomarkers, determining test reliability and reproducibility. Although many studies have considered inter-individual variation and technical variation introduced by experiment^13,14^, intra-individual transcriptional differences in normal breast tissue, as well as factors that contribute to intra-individual variability are not well characterized. Few studies of benign breast tissue have had sufficient resampling of tissues to allow assessment of whether a single biospecimen reliably represents the state of breast tissue. On the histologic level, we previously evaluated intra-individual variation in normal breast composition and found substantial variability in stromal and epithelial contents upon repeated sampling^6^. The impact of these histological differences on gene expression is not well characterized but is crucial for the reliability of transcriptional biomarkers.

This study employed a hierarchical sampling structure in the Normal Breast Study (NBS) to investigate intra-individual variation in benign breast gene expression. We analyzed the microarray gene expression data of 288 samples from 170 women using multiple samples per patient collected from different regions and separate sections of the breast. In addition, we identified participant or breast tissue histological characteristics that were associated with intra-individual variability.

## Materials and Methods

### Study population

The NBS is a study of breast cancer microenvironment and normal breast tissue conducted at UNC Hospitals in Chapel Hill, North Carolina. The details of study design and participant recruitment were published previously^15^. Briefly, participants were women ≥18 years who underwent breast surgery between October 2009 and April 2013, and consented to donate breast tissue. The NBS study population included 399 women with breast cancer and 75 women without malignant disease. All participants donated at least one histologically normal breast tissue specimen (as assessed by pathology assistants at UNC Hospitals). A telephone interview was conducted to collect demographic and breast cancer risk factor exposure data. Medical records were abstracted to obtain patients’ medical history, mammographic screening, breast cancer–related treatment, tumor pathology, and breast surgery data. All participants provided written informed consent, and all study protocols were approved by the UNC School of Medicine’s Institutional Review Board. All methods of data collection and analysis were performed in accordance with relevant guidelines and regulations with appropriate quality control.

**Figure 1:**
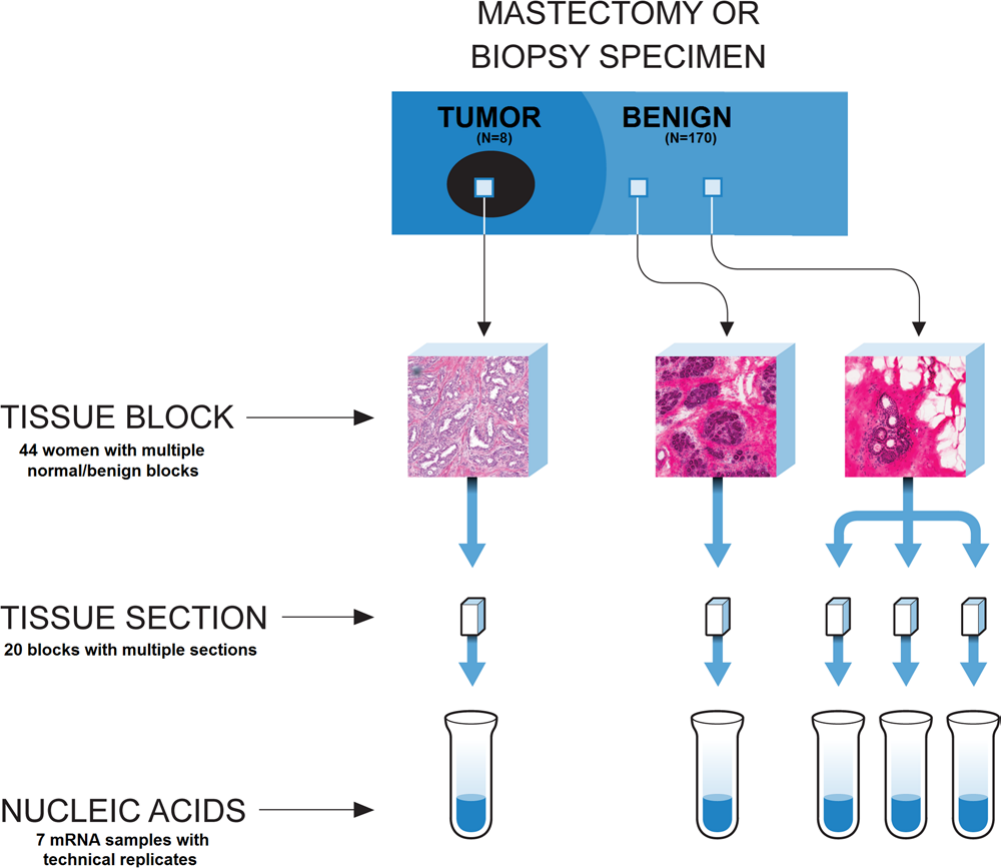
Sampling structure of the UNC Normal Breast Study.

This analysis included 288 normal samples from 170 women of the NBS with gene expression data. Our primary intra-individual analysis was based on 57 women having multiple normal samples. To study gene expression variability at different levels, we employed the NBS hierarchical sampling structure: one or more blocks were sampled from each participant; one or more sections were sampled from each block; and mRNA expression was measured from each section with some mRNAs run twice on microarray platform (technical replicates). We also collected tumor gene expression data from a small number of breast cancer patients (n = 8) to qualitatively compare variability observed in normal tissue to that observed in tumors. The data structure, including number of normal samples per woman, is shown in Supplementary Table 1 and summarized in Fig. 1.

### Tissue sectioning and image analysis

For each tissue block, sections of alternating width (100 µm and 20 μm) were cut over dry ice for histological and gene expression analyses. The 100 µm sections were used for RNA isolation as described below, while the 20-μm sections were stained with hematoxylin and eosin (H&E) and used for high-resolution scanning and histological composition annotation. The details of histological annotation by Aperio Scan-Scope XT Slide Scanner and Genie Classifier have been published previously^3,6,15^. Standard, validated algorithms were used to partition epithelium, non-fatty stroma, and adipose tissue (in mm^2^) and identify the number of nuclei per unit area. The number of cells per epithelial, stromal, and adipose tissue area were calculated to represent cellular density (in cells/mm^2^). Variability in these composition parameters have been described previously^6^, and the current analysis utilized mean values across blocks and sections.

### mRNA isolation and microarrays

100 µm sections were homogenized as described previously^15^. mRNA was isolated following standard manufacturer protocols using RNeasy kits. The quality and quantity were analyzed on an Agilent 2100 Bioanalyzer and a ND-1000 NanoDrop spectrophotometer, respectively. Two-color 4 × 44 K Agilent whole-genome arrays (Version 1 or Version 2) were run on each mRNA samples, with the reference channel representing a strata gene Universal Human Reference RNA sample spiked with breast cancer cell line RNA (MCF-7 and ME16C) to increase expression of breast-specific genes. Expression data was preprocessed as follows: lowess-normalization, setting values of the probes that had a signal less than 10 dpi in either channel as missing, excluding probes that had more than 20% missing data across all samples, imputing missing values using k-nearest neighbors’ imputation (with k = 10), collapsing the replicate probes by averaging, and median-centering genes. A total of 12,429 genes passed the filters and were included in the analysis. Microarray data used in these analyses is publicly available through the Gene Expression Omnibus (under preparation).

### Statistical analysis

Using the hierarchical sampling strategy of multiple blocks per patient, multiple sections per block, and multiple technical replicates per section, we studied intra-individual gene expression variation at each level. We used the variation-by-distance (VD) metric, wherein the variation was estimated by the Euclidean distance between samples (details of VD calculation are provided in supplementary materials). Higher values of VD indicate higher variability. We compared VD at block, section, and technical replicate levels using t-tests. We used a nested ANOVA to obtain the proportion of intra-individual variability attributed to block and section levels^16^. Since intra-individual variation may differ by signatures/pathways, intra-individual variation of several previously published signatures (Supplementary Table 2), including composition-related signatures (epithelial signature^17^, stromal signature^17^, and immune signature)^18^, tumorigenesis-related signatures (p53 signature^19^, proliferation signature^20^, and hypoxia signature)^21^, and risk factor-related signatures (age signature^2^, and obesity signature^4^ and parity signature)^22^, were also evaluated using the same methods. To exclude potential confounding due to different version, variation was estimated among samples measured by a single version of microarray platform.

To identify patient factors associated with high intra-individual variability in breast gene expression, we classified patients as ‘heterogeneous’ if inter-block VD was greater than the inter-block median VD (n = 44), or if inter-section VD was above the inter-block median VD (n = 13 women who did not have multiple blocks). We estimated the associations between high intra-individual variability and individual characteristics (age, menopausal status, obesity, race, parity, oral conceptive, hormone replacement therapy) or tissue characteristics (tissue source, histological area and nuclear density in adipose and epithelium and stroma compartments) using Fisher’s exact tests. Statistical significance was defined as p < 0.05. All statistical analyses were performed using R, version 3.0.1.

## Results

### Intra-individual variability of global gene expression profile

**Figure 2:**
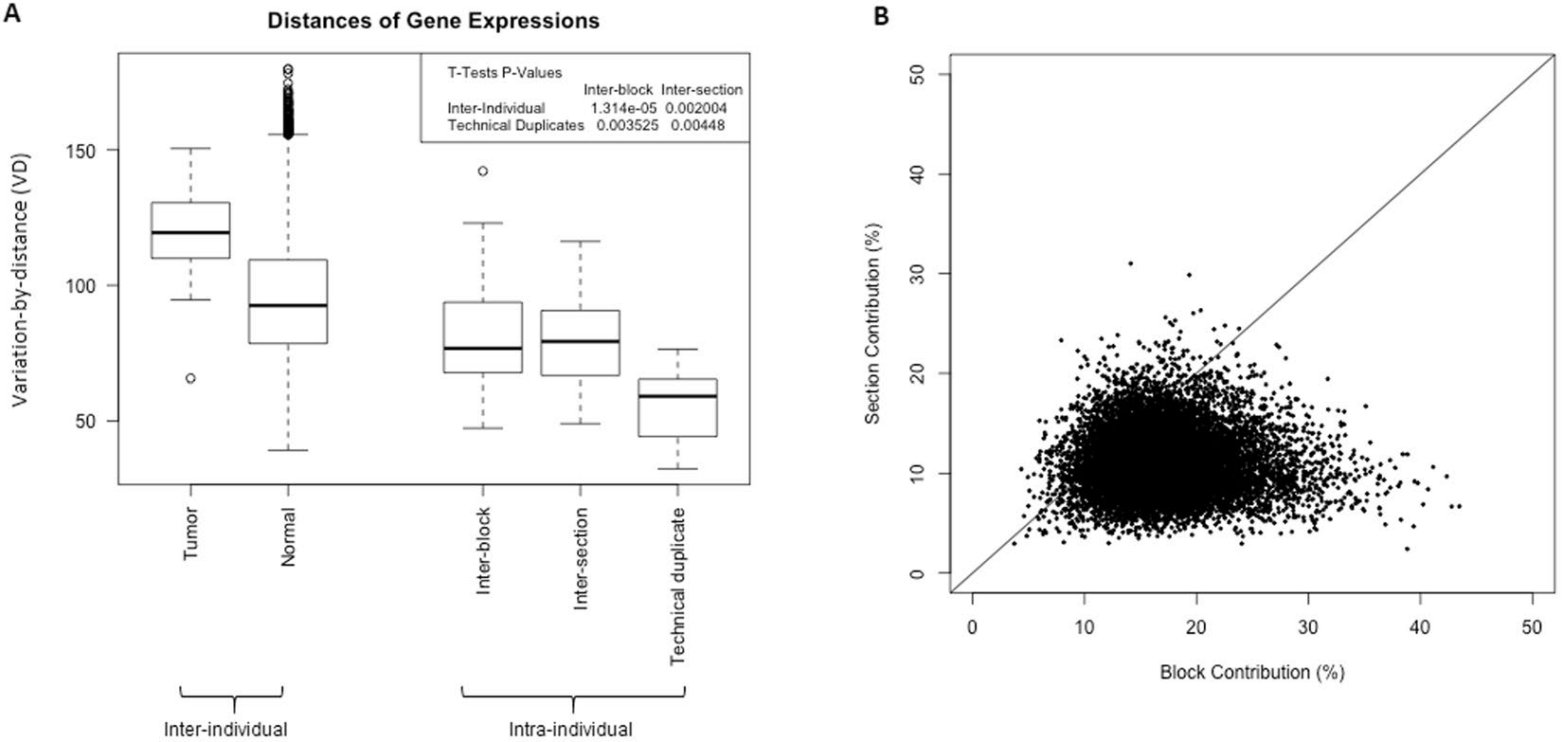
Global gene expression variability of histologically normal breast tissue. (**A**) Global gene expression variation at different levels. Y-axis is variation-by-distance (VD). Greater distance indicates higher variation. Inter-individual variability, n = 8 for tumor and n = 184 for normal; intra-individual variability, n = 49 for inter-block, n = 20 for inter-section, and n = 7 for technical duplicates. **(B)** Contribution of block and section to overall expression variation of genes. X- and Y-axes are percentage of contribution of block and section to the global gene expression variation, respectively. Each dot represents one gene. The diagonal solid line indicates the ideal situation where contribution of block and section is equal.

To assess global gene expression, we calculated intra-individual variability (variability across replicate blocks and sections), inter-individual variability (across women), and technical variability (using replicate microarrays on the same isolated mRNA samples) for our hierarchical samples of normal tissues, as well as inter-individual variability in tumor expression in a small set of samples. As shown in Fig. 2A, intra-individual variability (measured by VD) was lower than inter-individual variability, and significantly higher than the variability of technical replication (t-tests p < 0.01). Although block-level variation in global gene expression did not show significant difference from section-level variation, in the variation contribution analysis (Fig. 2B), blocks appeared to explain higher percentage of intra-individual variation than sections. In addition, we observed the percentage of intra-individual variation explained by block and section differed from gene-to-gene, accounting up to 40% in some specific genes. We therefore assessed how intra-individual variability impacted several multi-gene signatures.

### Intra-individual variability of the selected transcriptional signatures

**Figure 3:**
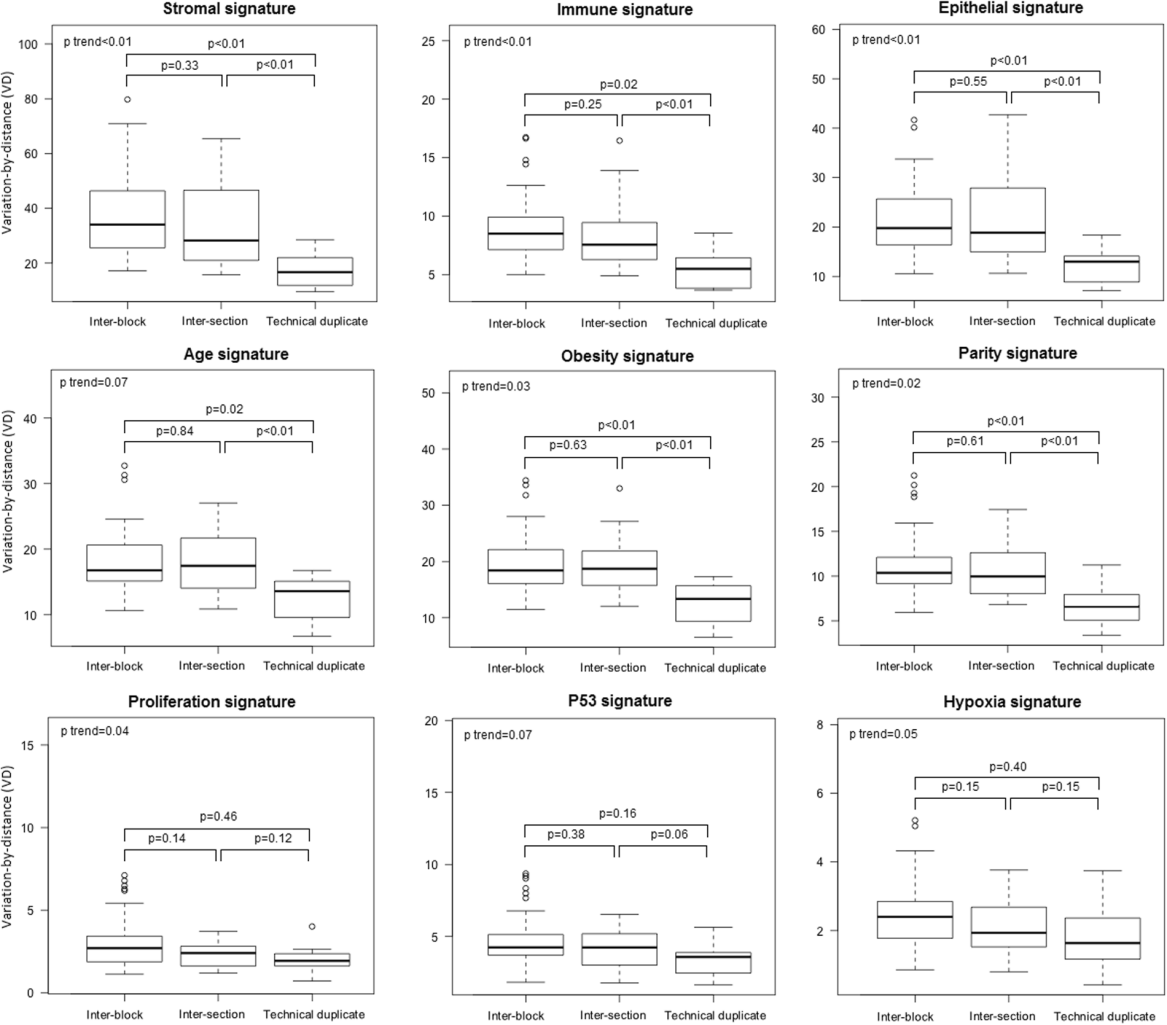
Gene expression variation of selected signatures.

We assessed the intra-individual variability of several selected multi-gene signatures, including previously published risk factor-related signatures (age, obesity, and parity), tissue composition-related signatures (stroma, immune, and epithelium), and tumorigenesis-related signatures (proliferation, p53, and hypoxia). As shown in Fig. 3, intra-individual variability was different depending upon the specific signature. Risk factor-associated signatures showed a similar pattern that was observed in global gene expression, with the lowest variability in technical replicates (block vs. technical replicate p < 0.01, section vs. technical replicate p < 0.01) and similar variability at block and section levels. For tissue composition-associated signatures, intra-individual variability was suggestively associated with spatial distance (p < 0.01 for trend test of variability from technical replicate to inter-section, and to inter-block), but tumorigenesis-associated signatures had low intra-individual variation, with inter-section and inter-block variations not statistically distinct from technical replicates.

### Factors associated with intra-individual variability

To identify patient factors associated with intra-individual variability in gene expression, we categorized individuals as heterogeneous or homogeneous based on global gene expression, and evaluated this categorical variable in association with demographic and histologic characteristics (Table 1). Higher stromal density (OR = 3.42, 95% CI = 1.15–10.15) and low stroma area (OR = 0.29, 95% CI = 0.10–0.86) were significantly associated with intra-individual variability. These associations remained after adjusting for age (stroma density, adjusted OR = 3.25, 95% CI = 1.08–9.74; stroma area, adjusted OR = 0.31, 95% CI = 0.10–0.94) or BMI (stroma density, adjusted OR = 3.09, 95% CI = 1.01–9.48; stroma area, adjusted OR = 0.32, 95% CI = 0.10–0.96). Moreover, young age (<50 years, OR = 2.44, 95% CI = 0.79–7.51), lower BMI (<30 kg/m^2^, OR = 1.97, 95% CI = 0.65–5.95), and lower adipose density (OR = 1.90, 95% CI = 0.66–5.46) were suggested to be associated with increased intra-individual variation in gene expression profiles. These factors were also evaluated in the intra-individual variability of the selected transcriptional signatures. Similar to patterns for global expression, age and breast composition were associated with intra-individual variability.

**Table 1:** Characteristics and intra-individual variability in histological normal breast tissue.

	All (n = 57) n(col%)	Homogeneous (n = 28) n(col%)	Heterogeneous (n = 29) n(col%)	OR (95% CI)	*P* ^a^
Age, year					
≥50	37 (64.91)	21 (75)	16 (55.17)	1	0.1665
<50	20 (35.09)	7 (25)	13 (44.83)	2.44 (0.79, 7.51)	
Menopausal status					
Postmenopausal	39 (69.64)	20 (71.43)	19 (67.86)	1	1
Premenopausal	17 (30.36)	8 (28.57)	9 (32.14)	1.18 (0.38, 3.71)	
BMI					
Non-obese	37 (64.91)	16 (57.14)	21 (72.41)	1	0.2744
Obese	20 (35.09)	12 (42.86)	8 (27.59)	0.51 (0.17, 1.54)	
Race					
White	33 (60.00)	16 (59.26)	17 (60.71)	1	1
Black	22 (40.00)	11 (40.74)	11 (39.29)	0.94 (0.32, 2.77)	
Parity					
Nulliparous	10 (18.87)	5 (18.52)	5 (19.23)	1	1
Parous	43 (81.13)	22 (81.48)	21 (80.77)	0.95 (0.24, 3.78)	
OC					
No	9 (16.98)	4 (14.81)	5 (19.23)	1	0.7277
Yes	44 (83.02)	23 (85.19)	21 (80.77)	0.73 (0.17, 3.09)	
HRT					
No	23 (57.50)	12 (60.00)	11 (55.00)	1	1
Yes	17 (42.50)	8 (40.00)	9 (45.00)	1.23 (0.35, 4.31)	
Tissue source					
Lumpectomy or Mastectomy	36 (73.47)	21 (80.77)	15 (65.22)	1	0.3320
Reduction	13 (26.53)	5 (19.23)	8 (34.78)	2.24 (0.61, 8.21)	
Adipose density^b^, cells/mm^2^					
≤median	27 (47.37)	11 (39.29)	16 (55.17)	1	0.2924
>median	30 (52.63)	17 (60.71)	13 (44.83)	0.53 (0.18, 1.51)	
Epithelium density^b^, cells/mm^2^					
≤median	32 (56.14)	17 (60.71)	15 (51.72)	1	0.5964
>median	25 (43.86)	11 (39.29)	14 (48.28)	1.44 (0.50, 4.13)	
Stroma density^b^, cells/mm^2^					
≤median	28 (49.12)	18 (64.29)	10 (34.48)	1	0.0348
>median	29 (50.88)	10 (35.71)	19 (65.52)	3.42 (1.15, 10.15)	
Adipose area^b^, mm^2^					
≤median	32 (56.14)	14 (50.00)	18 (62.07)	1	0.4287
>median	25 (43.86)	14 (50.00)	11 (37.93)	0.61 (0.21, 1.75)	
Epithelium area^b^, mm^2^					
≤median	22 (38.60)	9 (32.14)	13 (44.83)	1	0.4173
>median	35 (61.40)	19 (67.86)	16 (55.17)	0.58 (0.20, 1.72)	
Stroma area^b^, mm^2^					
≤median	27 (47.37)	9 (32.14)	18 (62.07)	1	0.0343
>median	30 (52.63)	19 (67.86)	11 (37.93)	0.29 (0.10, 0.86)	

^a^P values of Fisher exact tests. Total numbers vary due to missing.

^b^Adipose density median = 237 cells/mm^2^, epithelium density median = 5,501 cells/mm^2^, stroma density median = 1,360 cells/mm^2^, adipose area median = 53 mm^2^, epithelium area median = 10 mm^2^, stroma area = 34 mm^2^.

## Discussion

We evaluated intra-individual variation in benign breast gene expression using a hierarchical sampling scheme. We observed relatively high intra-individual reliability in global gene expression, despite that block-level variation was suggested to be slightly higher than section-level variation. However, the degree of intra-individual variability depended upon biological pathways/features, with composition-related signatures (e.g., stromal signature) showing a higher intra-individual variability than other biological-function specific pathways. We also observed that that histological tissue composition and key demographic variables (e.g., age, obesity) were associated with intra-individual variability in benign breast gene expression.

In the past decade, many studies have evaluated inter-individual gene expression in normal or cancer-adjacent tissue as a predictor of survival or in association with tumor characteristics or exposure history^4,9,10,17,22^. The reliability and reproducibility of these findings have yet to be well determined, and may depend, in part, on whether a single biospecimen procured at one point in time represents the underlying biology of interest. Increasing sample sizes is a straightforward way to tackle this issue, however, this approach is costly and must be driven by knowledge of how many samples are required to represent the relevant biology. Our findings have important implications for biospecimen sampling strategies, suggesting that for some signatures, a single sample may provide representative gene expression data. In particular, intra-individual variation was very low for proliferation, p53 and hypoxia signatures. Yet, we also observed that global gene expression and expression of composition-related signatures showed higher levels of variability between sections and blocks.

Our finding of high intra-individual variability of composition-related signatures is consistent with previous histological studies where histologic measures (e.g., stromal percent area and terminal duct lobular unit size) had a low/moderate agreement across different regions of the same normal breast tissue block^6,23–25^. It has also been previously reported that there is substantial variability of pathological and molecular characteristics within tumor tissue, according to cell mixture/tissue composition^26–29^. As an intrinsic feature, intra-tumor heterogeneity results from the dynamic evolution of tumor cells and their interaction with microenvironment, and plays an important role in breast cancer progression and therapy resistance^30–32^. For normal breast tissue, the extent to which intra-individual variability is an intrinsic feature of the breast, versus a reflection of a biospecimen sampling, has been poorly understood. To explore this question, we assessed the relationships between intra-individual heterogeneity in gene expression and several available woman/tissue characteristics. While these associations are largely insignificant, we did detect significant differences according to histological features including stromal nuclear density and area. Our results indicate that intra-individual variability may be strongly impacted by tissue composition. Besides histological features, we found that older and non-obese women tended to have breast tissue with more heterogeneous gene expression. Age and obesity are well-known risk factors for breast cancer and significantly affect breast tissue composition^15,17,33^, suggesting we cannot exclude the possibility that normal tissue heterogeneity is an intrinsic feature of breast, reflecting previous exposure history. Further study is needed to relate intra-individual heterogeneity to breast cancer risk or prognostic factors to clarify its intrinsicality and understand its biological significance in tumor development and progression. On the other hand, our results demonstrate that tissue composition may confound mRNA transcripts extracted from bulk normal tissue, particularly when normal tissue analysis is to ascertain more subtle effects of target phenotypes (e.g., breastfeeding) or epithelial cells are of interest.

Our findings should be interpreted in light of some limitations. Although multiple samples at different levels per patient provided a unique opportunity to study intra-individual variability, not all women donated samples at all levels, as sample availability was dependent on availability of normal tissue at time of surgery. Moreover, our study compiled histologically normal breast tissues from women undergoing a variety of procedures. Previous research suggests that cancer-adjacent tissue possesses some differences from benign tissues of disease-free women^9,34–36^. To test the potential impact of the samples from breast cancer patients, we conducted a series of sensitivity analysis: (1) checking the similarity of gene expression by tissue sources using principle component analysis (Supplementary Figure 1A); (2) re-evaluating the variabilities across different levels after excluding tissue samples with distance to tumor less than 1 cm (n = 15, Supplementary Figure 1B); (3) comparing the intra-individual variabilities between the whole samples (n = 57) and the subset (n = 42) (Supplementary Figure 1B). We did not observe remarkable changes in these sensitivity analyses. Therefore, we do not think the potential biological alterations in cancer-adjacent tissue will change the conclusions in our study significantly. Last, our sample size hampered the precision of our estimates to evaluate the association between intra-individual variation and breast cancer risk factors.

In summary, our study characterized intra-individual variation in gene expression of normal breast tissues, both globally and by selected transcriptional signatures. Our results underscore the importance of considering intra-individual variability in tissue-based biomarker development. Validation of our study findings in future studies is needed to further characterize heterogeneity in candidate histological and molecular biomarkers of breast cancer risk within normal breast tissue.

## Electronic supplementary material

Supplementary information
