# Supplementary material for: Intra-individual Gene Expression Variability of Histologically Normal Breast Tissue
Source: Sci Rep. 2018 Jun 14;8:9137. doi: 10.1038/s41598-018-27505-y (PMC6002361; doi:10.1038/s41598-018-27505-y)
Supplement: Supplementary file 1 — Supplementary information [file 41598_2018_27505_MOESM1_ESM.pdf]

Intra-individual Gene Expression Variability of Histologically Normal Breast Tissue

Xuezheng Sun*[1,2], Yue Shan[3], Quefeng Li[3], Lynn Chollet-Hinton[4], Erin L. Kirk[1], Gretchen L. Gierach[5], Melissa A. Troester[1, 2, 4]

1. Department of Epidemiology, Gillings School of Public Health, University of North Carolina at Chapel Hill.
2. Center for Environmental Health and Susceptibility, University of North Carolina at Chapel Hill.
3. Department of Biostatistics, Gillings School of Public Health, University of North Carolina at Chapel Hill.
4. Lineberger Comprehensive Cancer Center, University of North Carolina at Chapel Hill.
5. Metabolic Epidemiology Branch, Division of Cancer Epidemiology & Genetics, National Cancer Institute

Supplementary Material

Definition of Variation-by-distance (VD): We decompose the gene expression through a hierarchical structure and quantify the variation at each hierarchy by a distance-based measurement. Let $h = 1,2,3,4$ denote the hierarchies of individual, block, section, and technical replicate, respectively. For the g-th gene, let $c_{h_i,g}$ denote the average expression of the g-th gene for the i-th object in the h-th hierarchy. For $h \in \{1,2,3\}$, we have $c_{h_i,g} = \frac{1}{\left|\Omega_{h_i,g}\right|}\sum_{k\in\Omega_{h_i,g}} c_{k,g}$, where Ω_{h_i} denotes the index set at the $(h+1)$-th hierarchy that contains the h_i-th object, and $\left|\Omega_{h_i,g}\right|$ denotes the size of set $\Omega_{h_i,g}$. For $h = 4,$ $c_{h_i,g}$ is the raw expression of the g-th gene for the h_i-th object. For a total of G genes, the Euclidean distance between the h_i-th object and the h_j-th object is defined as

$$d\left(c_{h_i}, c_{h_j}\right) = \sqrt{\sum_{g=1}^{G}\left(c_{h_i,g} - c_{h_j,g}\right)^2}$$

Then, we measured the variation of the h_i-th object by the Euclidean distance between any pair of its appearance at the $(h+1)$-th hierarchy. We named such a quantity as the *variation-by-distance* for the h_i-th object.

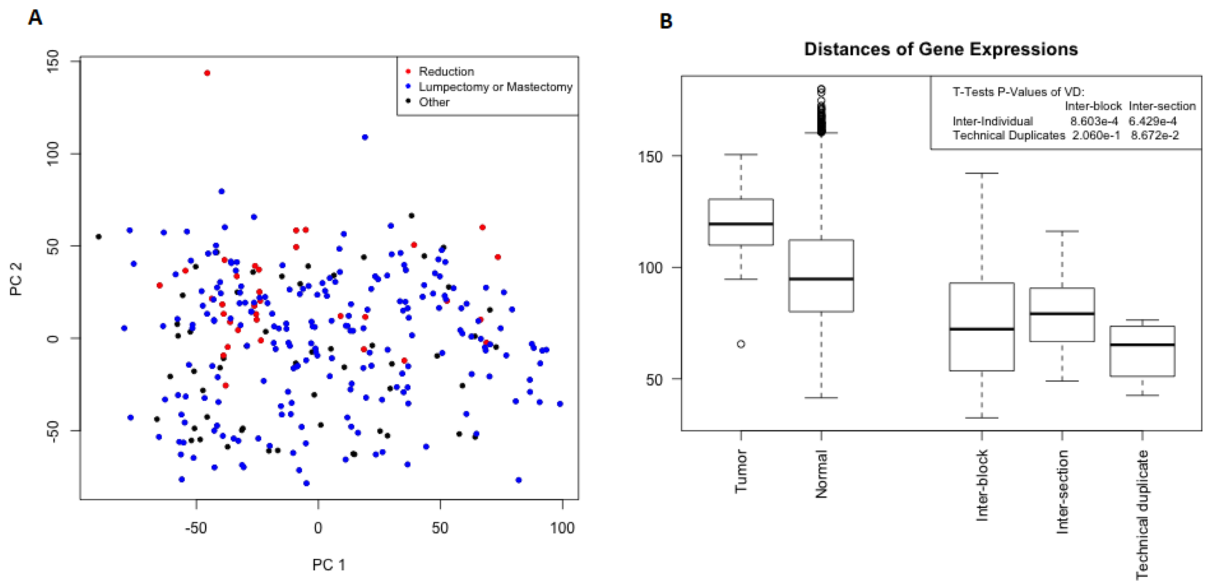

Supplementary Figure 1. Results of sensitivity analyses. (A) Principle component analysis plot by tissue sources. **(B)** Global gene expression variation at different levels in the subset (n=42) excluding samples with distance to tumor less than 1 cm. Y-axis is variation-by-distance (VD). Greater distance indicates higher variation. For inter-individual variability, n = 8 for tumor and n = 164 for normal; for intra-individual variability, n = 23 for inter-block, n = 20 for inter-section, and n=4 for technical duplicates. The block-level variabilities in all samples (n=57, Figure 2A) and in the subset (n=42) (section-level variability was not compared because the exclusion did not change the sample numbers at that level) were compared using paired permutation test, and no significant difference was detected (p-value =0.0943).

Supplementary Table 1 Samples included at various levels.

Patient_ID	Tissue_ID	Block_ID	Tissue_type	Array_version	Section	Technical_replicate
NBS09012	94921251	2	Tumor	V1	0	0
NBS10027	96930011	1	Tumor	V1	0	0
NBS10029	99704033	5	Tumor	V1	0	0
NBS10038	98612773	3	Tumor	V1	0	0
NBS10046	90681222	3	Tumor	V1	0	0
NBS10047	97710537	2	Tumor	V1	0	0
NBS10051	90010372	4	Tumor	V1	0	0
NBS10080	92839463	1	Tumor	V1	0	0
NBS09010	98627409	2	Cancer Adjacent Normal	V2	0	0
NBS09012	94322229	2	Cancer Adjacent Normal	V1	0	0
NBS09012	94322229	2	Cancer Adjacent Normal	V2	1	0
NBS09012	94322229	3	Cancer Adjacent Normal	V1	0	1
NBS09012	94322229	3	Cancer Adjacent Normal	V1	0	1
NBS09012	94322229	3	Cancer Adjacent Normal	V1	0	1
NBS09015	90337585	2	Cancer Adjacent Normal	V2	0	0
NBS09018	91271643	2	Others	V2	1	0
NBS10027	99519134	2	Cancer Adjacent Normal	V1	0	0
NBS10027	99519134	5	Cancer Adjacent Normal	V1	0	0
NBS10029	93280626	2	Cancer Adjacent Normal	V1	0	0
NBS10029	93280626	3	Cancer Adjacent Normal	V1	0	0

NBS10030	97636476	2	Cancer Adjacent Normal	V1	0	0
NBS10030	97636476	3	Cancer Adjacent Normal	V1	0	0
NBS10030	97636476	3	Cancer Adjacent Normal	V2	1	0
NBS10032	90178070	4	Cancer Adjacent Normal	V2	1	0
NBS10036	91406728	6	Cancer Adjacent Normal	V2	0	0
NBS10037	95786794	2	Cancer Adjacent Normal	V2	0	0
NBS10038	95518494	2	Cancer Adjacent Normal	V1	0	1
NBS10038	95518494	2	Cancer Adjacent Normal	V1	0	1
NBS10038	95518494	2	Cancer Adjacent Normal	V2	0	0
NBS10038	95518494	4	Cancer Adjacent Normal	V1	0	0
NBS10038	95518494	9	Cancer Adjacent Normal	V1	0	0
NBS10038	95518494	9	Cancer Adjacent Normal	V2	1	0
NBS10041	92220789	2	Others	V2	0	0
NBS10046	92384106	7	Cancer Adjacent Normal	V1	0	0
NBS10046	92384106	9	Cancer Adjacent Normal	V1	0	0
NBS10047	95644548	2	Cancer Adjacent Normal	V1	0	0

NBS10047	95644548	2	Cancer Adjacent Normal	V1	0	1
NBS10047	95644548	2	Cancer Adjacent Normal	V1	0	1
NBS10047	95644548	8	Cancer Adjacent Normal	V1	0	0
NBS10047	95644548	8	Cancer Adjacent Normal	V2	1	0
NBS10049	98717911	2	Cancer Adjacent Normal	V1	0	0
NBS10049	98717911	4	Cancer Adjacent Normal	V1	0	0
NBS10049	98717911	6	Cancer Adjacent Normal	V1	0	0
NBS10049	98717911	9	Cancer Adjacent Normal	V1	0	0
NBS10051	98684327	2	Cancer Adjacent Normal	V1	0	0
NBS10051	98684327	2	Cancer Adjacent Normal	V2	0	0
NBS10051	98684327	9	Cancer Adjacent Normal	V1	0	0
NBS10051	98684327	9	Cancer Adjacent Normal	V2	0	0
NBS10051	98684327	9	Cancer Adjacent Normal	V2	1	0
NBS10053	95184032	9	Cancer Adjacent Normal	V2	0	0
NBS10055	93071371	2	Cancer Adjacent Normal	V2	0	0

NBS10058	91068346	2	Cancer Adjacent Normal	V2	0	0
NBS10059	99792327	2	Cancer Adjacent Normal	V2	0	1
NBS10059	99792327	8	Cancer Adjacent Normal	V2	1	0
NBS10060	94482775	8	Cancer Adjacent Normal	V2	0	0
NBS10062	96209762	2	Cancer Adjacent Normal	V2	0	0
NBS10063	97619225	2	Cancer Adjacent Normal	V2	0	0
NBS10063	97619225	9	Cancer Adjacent Normal	V2	0	0
NBS10063	97619225	9	Cancer Adjacent Normal	V2	1	0
NBS10065	94612124	8	Cancer Adjacent Normal	V2	0	0
NBS10067	92362565	2	Others	V2	0	0
NBS10067	92362565	3	Others	V2	0	0
NBS10069	91810705	8	Cancer Adjacent Normal	V2	1	0
NBS10072	97227730	2	Cancer Adjacent Normal	V1	0	0
NBS10072	97227730	6	Cancer Adjacent Normal	V1	0	0
NBS10072	97227730	8	Cancer Adjacent Normal	V1	0	0
NBS10073	96202213	2	Cancer Adjacent Normal	V2	0	1
NBS10073	96202213	2	Cancer Adjacent Normal	V2	0	0

NBS10074	92042407	1	Cancer Adjacent Normal	V2	0	0
NBS10077	95029484	2	Cancer Adjacent Normal	V1	0	0
NBS10077	95029484	2	Cancer Adjacent Normal	V2	1	0
NBS10077	95029484	4	Cancer Adjacent Normal	V1	0	0
NBS10077	95029484	6	Cancer Adjacent Normal	V1	0	0
NBS10077	95029484	8	Cancer Adjacent Normal	V1	0	0
NBS10077	95029484	8	Cancer Adjacent Normal	V2	1	0
NBS10080	95475745	2	Cancer Adjacent Normal	V1	0	0
NBS10080	95475745	2	Cancer Adjacent Normal	V2	1	0
NBS10080	95475745	8	Cancer Adjacent Normal	V1	0	0
NBS10080	95475745	8	Cancer Adjacent Normal	V2	1	0
NBS10080	95475745	8	Cancer Adjacent Normal	V2	1	0
NBS10081	99274342	2	Cancer Adjacent Normal	V1	0	0
NBS10081	99274342	4	Cancer Adjacent Normal	V1	0	0
NBS10081	99274342	6	Cancer Adjacent Normal	V1	0	0

NBS10081	99274342	9	Cancer Adjacent Normal	V1	0	0
NBS10081	99274342	9	Cancer Adjacent Normal	V2	1	0
NBS10082	91224048	2	Reduction	V2	0	0
NBS10082	91224048	2	Reduction	V2	1	0
NBS10083	91543579	3	Cancer Adjacent Normal	V2	0	1
NBS10084	98879026	4	Cancer Adjacent Normal	V1	0	0
NBS10084	98879026	6	Cancer Adjacent Normal	V1	0	0
NBS10084	98879026	8	Cancer Adjacent Normal	V1	0	0
NBS10088	98415342	1	Cancer Adjacent Normal	V2	0	0
NBS10089	93309698	2	Others	V2	0	1
NBS10090	96590005	8	Cancer Adjacent Normal	V2	0	0
NBS10093	90915042	1	Cancer Adjacent Normal	V2	0	0
NBS10094	90668096	5	Cancer Adjacent Normal	V2	0	0
NBS10095	94183647	2	Cancer Adjacent Normal	V2	0	0
NBS10096	90900150	2	Cancer Adjacent Normal	V2	0	0
NBS10097	97519847	2	Reduction	V2	0	0
NBS10097	97519847	2	Reduction	V2	1	0
NBS10098	94152899	2	Cancer Adjacent Normal	V2	0	0
NBS10099	91922195	2	Reduction	V2	0	0

NBS10100	93627867	2	Cancer Adjacent Normal	V1	0	0
NBS10100	93627867	4	Cancer Adjacent Normal	V1	0	0
NBS10100	93627867	6	Cancer Adjacent Normal	V1	0	0
NBS10100	93627867	8	Cancer Adjacent Normal	V1	0	0
NBS10100	93627867	8	Cancer Adjacent Normal	V2	1	0
NBS10103	94696606	1	Others	V2	0	0
NBS10104	90158635	2	Cancer Adjacent Normal	V2	1	1
NBS10105	91951624	2	Others	V2	0	0
NBS10105	91951624	5	Others	V2	0	0
NBS10106	98708837	1	Cancer Adjacent Normal	V2	0	0
NBS10107	90998329	2	Cancer Adjacent Normal	V1	0	0
NBS10107	90998329	4	Cancer Adjacent Normal	V1	0	0
NBS10107	90998329	6	Cancer Adjacent Normal	V1	0	0
NBS10107	90998329	8	Cancer Adjacent Normal	V1	0	0
NBS10107	90998329	8	Cancer Adjacent Normal	V2	1	0
NBS10109	92171917	0	Cancer Adjacent Normal	V2	0	0
NBS10110	96468624	2	Cancer Adjacent Normal	V1	0	0

NBS10110	96468624	2	Cancer Adjacent Normal	V2	1	0
NBS10110	96468624	6	Cancer Adjacent Normal	V1	0	0
NBS10110	96468624	9	Cancer Adjacent Normal	V1	0	0
NBS10110	96468624	9	Cancer Adjacent Normal	V2	1	0
NBS10116	99556326	1	Cancer Adjacent Normal	V2	0	0
NBS10117	95620571	2	Reduction	V2	0	0
NBS10117	95620571	4	Reduction	V2	1	0
NBS10118	96454897	2	Cancer Adjacent Normal	V1	0	0
NBS10118	96454897	4	Cancer Adjacent Normal	V1	0	0
NBS10118	96454897	6	Cancer Adjacent Normal	V1	0	0
NBS10118	96454897	8	Cancer Adjacent Normal	V1	0	0
NBS10118	96454897	8	Cancer Adjacent Normal	V2	0	0
NBS10121	93203917	1	Cancer Adjacent Normal	V2	0	0
NBS10123	95950937	1	Cancer Adjacent Normal	V2	0	1
NBS10125	99220113	8	Cancer Adjacent Normal	V2	0	0
NBS10126	96503198	8	Cancer Adjacent Normal	V2	0	0
NBS10128	98660566	2	Cancer Adjacent Normal	V2	0	0

NBS10128	98660566	9	Cancer Adjacent Normal	V2	0	0
NBS10129	92367044	2	Cancer Adjacent Normal	V1	0	0
NBS10129	92367044	4	Cancer Adjacent Normal	V1	0	0
NBS10129	92367044	6	Cancer Adjacent Normal	V1	0	0
NBS10129	92367044	8	Cancer Adjacent Normal	V1	0	0
NBS10129	92367044	8	Cancer Adjacent Normal	V2	1	0
NBS10131	98340219	2	Others	V2	0	0
NBS10132	90252966	2	Reduction	V2	0	0
NBS10132	90252966	2	Reduction	V2	1	0
NBS10133	98085434	2	Cancer Adjacent Normal	V1	0	0
NBS10133	98085434	4	Cancer Adjacent Normal	V1	0	0
NBS10133	98085434	6	Cancer Adjacent Normal	V1	0	0
NBS10133	98085434	8	Cancer Adjacent Normal	V1	0	0
NBS10134	90679705	4	Cancer Adjacent Normal	V1	0	0
NBS10134	90679705	6	Cancer Adjacent Normal	V1	0	0
NBS10134	90679705	8	Cancer Adjacent Normal	V1	0	0
NBS10135	92272293	1	Others	V2	0	0
NBS10141	99988438	8	Cancer Adjacent Normal	V2	0	0

NBS10142	96245626	2	Others	V2	0	0
NBS10143	92877471	8	Cancer Adjacent Normal	V2	1	0
NBS10145	93223865	4	Cancer Adjacent Normal	V2	0	0
NBS10146	96186002	8	Cancer Adjacent Normal	V2	0	0
NBS10148	97208243	8	Cancer Adjacent Normal	V2	0	0
NBS10149	95130985	2	Cancer Adjacent Normal	V2	0	1
NBS10151	95801551	2	Cancer Adjacent Normal	V1	0	0
NBS10151	95801551	4	Cancer Adjacent Normal	V1	0	0
NBS10151	95801551	6	Cancer Adjacent Normal	V1	0	0
NBS10151	95801551	8	Cancer Adjacent Normal	V1	0	0
NBS10152	92998483	2	Cancer Adjacent Normal	V1	0	0
NBS10152	92998483	4	Cancer Adjacent Normal	V1	0	0
NBS10152	92998483	6	Cancer Adjacent Normal	V1	0	0
NBS10152	92998483	8	Cancer Adjacent Normal	V1	0	0
NBS10152	92998483	8	Cancer Adjacent Normal	V2	1	1
NBS10154	90860750	2	Others	V2	0	0
NBS10155	96533807	2	Cancer Adjacent Normal	V2	0	0

NBS10158	94865557	2	Cancer Adjacent Normal	V2	0	0
NBS10159	91081125	2	Cancer Adjacent Normal	V2	0	0
NBS10161	96935374	2	Reduction	V2	0	0
NBS10161	96935374	2	Reduction	V2	0	1
NBS10162	96771944	2	Reduction	V2	0	0
NBS10162	96771944	2	Reduction	V2	1	0
NBS10162	96771944	4	Reduction	V2	0	0
NBS10164	97383111	2	Cancer Adjacent Normal	V2	0	0
NBS10164	97383111	2	Cancer Adjacent Normal	V2	1	0
NBS10165	94543204	2	Reduction	V2	0	0
NBS10165	94543204	2	Reduction	V2	1	0
NBS10165	94543204	2	Reduction	V2	1	0
NBS10166	93211407	1	Cancer Adjacent Normal	V2	0	0
NBS10167	95275913	2	Cancer Adjacent Normal	V2	1	1
NBS10169	92556307	2	Cancer Adjacent Normal	V2	0	0
NBS10169	92556307	2	Cancer Adjacent Normal	V2	1	0
NBS10170	96136254	8	Cancer Adjacent Normal	V2	0	0
NBS10174	97842975	8	Cancer Adjacent Normal	V2	1	0
NBS10175	90633538	1	Others	V2	0	0
NBS10176	93168235	8	Cancer Adjacent Normal	V2	0	0
NBS10179	98951288	8	Cancer Adjacent Normal	V2	1	0

NBS10180	98048572	8	Cancer Adjacent Normal	V2	0	0
NBS10181	94349438	8	Cancer Adjacent Normal	V2	0	0
NBS10181	94349438	8	Cancer Adjacent Normal	V2	1	0
NBS10188	92720820	8	Cancer Adjacent Normal	V2	0	0
NBS11191	99541583	8	Cancer Adjacent Normal	V2	0	0
NBS11193	99777039	9	Cancer Adjacent Normal	V2	0	0
NBS11195	99254690	2	Cancer Adjacent Normal	V2	0	0
NBS11197	95342515	2	Reduction	V2	0	0
NBS11197	95342515	2	Reduction	V2	1	0
NBS11198	95714861	8	Cancer Adjacent Normal	V2	0	1
NBS11200	92642727	8	Cancer Adjacent Normal	V2	0	0
NBS11201	92921469	2	Reduction	V2	0	0
NBS11201	92921469	4	Reduction	V2	0	0
NBS11204	92611805	8	Cancer Adjacent Normal	V2	1	0
NBS11206	92614429	2	Reduction	V2	0	0
NBS11206	92614429	2	Reduction	V2	1	0
NBS11207	90415522	8	Cancer Adjacent Normal	V2	0	0
NBS11208	91418350	2	Others	V2	0	0
NBS11209	97705206	8	Cancer Adjacent Normal	V2	0	0
NBS11210	98807324	2	Cancer Adjacent Normal	V2	0	1

NBS11214	93854651	2	Cancer Adjacent Normal	V2	0	0
NBS11218	92357102	8	Cancer Adjacent Normal	V2	0	0
NBS11220	98256464	8	Cancer Adjacent Normal	V2	0	0
NBS11221	91941765	1	Cancer Adjacent Normal	V2	0	0
NBS11229	95524989	8	Cancer Adjacent Normal	V2	0	0
NBS11233	90457284	10	Others	V2	0	0
NBS11236	92979756	8	Cancer Adjacent Normal	V2	1	0
NBS11237	90375635	2	Cancer Adjacent Normal	V2	0	0
NBS11239	94963998	2	Reduction	V2	1	0
NBS11239	94963998	2	Reduction	V2	1	0
NBS11240	93278828	2	Reduction	V2	0	0
NBS11240	93278828	2	Reduction	V2	1	0
NBS11241	90509555	8	Cancer Adjacent Normal	V2	0	0
NBS11245	93859536	2	Cancer Adjacent Normal	V2	0	0
NBS11248	97705347	1	Cancer Adjacent Normal	V2	0	0
NBS11251	94435138	8	Cancer Adjacent Normal	V2	0	0
NBS11252	90684911	1	Cancer Adjacent Normal	V2	0	1
NBS11256	90088402	2	Reduction	V2	0	0
NBS11256	90088402	2	Reduction	V2	1	0
NBS11261	90240128	2	Others	V2	0	0

NBS11262	96633128	2	Cancer Adjacent Normal	V2	0	0
NBS11262	96633128	8	Cancer Adjacent Normal	V2	0	0
NBS11262	96633128	8	Cancer Adjacent Normal	V2	1	0
NBS11265	99386831	8	Cancer Adjacent Normal	V2	0	0
NBS11272	91511352	1	Cancer Adjacent Normal	V2	0	1
NBS11276	93023968	2	Cancer Adjacent Normal	V2	0	0
NBS11276	93023968	4	Cancer Adjacent Normal	V2	0	0
NBS11285	99094021	8	Cancer Adjacent Normal	V2	0	0
NBS11287	95807681	1	Cancer Adjacent Normal	V2	0	1
NBS11297	98716335	2	Cancer Adjacent Normal	V2	0	0
NBS11297	98716335	8	Cancer Adjacent Normal	V2	1	1
NBS11297	98716335	8	Cancer Adjacent Normal	V2	0	0
NBS11299	93144020	4	Cancer Adjacent Normal	V2	0	1
NBS11300	99662678	8	Cancer Adjacent Normal	V2	0	0
NBS11301	93807204	2	Reduction	V2	0	0
NBS11309	99494965	2	Cancer Adjacent Normal	V2	0	0

NBS11309	99494965	8	Cancer Adjacent Normal	V2	0	0
NBS11310	91471011	2	Reduction	V2	0	1
NBS11311	95181889	4	Cancer Adjacent Normal	V2	0	1
NBS11316	92676253	2	Cancer Adjacent Normal	V2	0	1
NBS11316	92676253	2	Cancer Adjacent Normal	V2	0	1
NBS11317	98522964	8	Cancer Adjacent Normal	V2	0	0
NBS11320	90019498	8	Cancer Adjacent Normal	V2	0	0
NBS11321	95535928	8	Cancer Adjacent Normal	V2	0	0
NBS11325	96008594	2	Cancer Adjacent Normal	V2	0	1
NBS11325	96008594	4	Cancer Adjacent Normal	V2	0	0
NBS11326	90247669	8	Cancer Adjacent Normal	V2	0	1
NBS11331	95289435	2	Cancer Adjacent Normal	V2	0	0
NBS11331	95289435	8	Cancer Adjacent Normal	V2	0	0
NBS11331	95289435	8	Cancer Adjacent Normal	V2	1	0
NBS11345	90466491	8	Cancer Adjacent Normal	V2	0	0
NBS11349	97838650	8	Cancer Adjacent Normal	V2	0	0

NBS11354	95713137	8	Cancer Adjacent Normal	V2	0	0
NBS11356	97128227	2	Cancer Adjacent Normal	V2	0	0
NBS11357	90321670	8	Cancer Adjacent Normal	V2	0	0
NBS12374	91016089	2	Cancer Adjacent Normal	V2	0	0
NBS12374	91016089	8	Cancer Adjacent Normal	V2	0	0
NBS12376	91131474	1	Cancer Adjacent Normal	V2	0	1
NBS12377	91154260	1	Cancer Adjacent Normal	V2	0	1
NBS12378	90191529	2	Reduction	V2	0	0
NBS12378	90191529	2	Reduction	V2	1	0
NBS12384	96000658	8	Cancer Adjacent Normal	V2	0	0
NBS12387	91714162	2	Cancer Adjacent Normal	V2	0	0
NBS12387	91714162	8	Cancer Adjacent Normal	V2	0	0
NBS12392	96867650	2	Others	V2	0	0
NBS12393	99676918	2	Cancer Adjacent Normal	V2	0	0
NBS12393	99676918	8	Cancer Adjacent Normal	V2	0	0
NBS12394	97357321	2	Cancer Adjacent Normal	V2	0	0
NBS12394	97357321	8	Cancer Adjacent Normal	V2	0	0

NBS12395	97734107	2	Cancer Adjacent Normal	V2	0	0
NBS12395	97734107	8	Cancer Adjacent Normal	V2	0	0
NBS12399	96361233	4	Cancer Adjacent Normal	V2	0	1
NBS12401	94733060	1	Cancer Adjacent Normal	V2	0	1
NBS12402	97315493	1	Cancer Adjacent Normal	V2	0	1
NBS12402	97315493	1	Cancer Adjacent Normal	V2	0	1
NBS12412	98047129	8	Cancer Adjacent Normal	V2	0	1
NBS12415	93403558	2	Cancer Adjacent Normal	V2	0	0
NBS12415	93403558	8	Cancer Adjacent Normal	V2	0	0
NBS12419	95293858	2	Cancer Adjacent Normal	V2	0	0
NBS12419	95293858	8	Cancer Adjacent Normal	V2	0	0
NBS12424	97412332	4	Cancer Adjacent Normal	V2	0	1
NBS12428	90139379	2	Reduction	V2	0	1
NBS12440	90918558	4	Cancer Adjacent Normal	V2	0	1
NBS12458	95136107	4	Cancer Adjacent Normal	V2	0	1

Supplementary Table 2 Entrez ID of the selected signature

Age	Obesity	Parity
28966	32	140
89797	51	199
26823	98	241
3717	185	272
8663	310	283
5337	683	306
7292	719	644
6335	734	669
6660	762	683
122616	781	719
149351	788	759
799	794	919
55366	858	929
1454	1066	940
50507	1160	945
8869	1318	952
2011	1389	962
8840	1438	963
5136	1580	968
1154	1634	1029
5195	1879	1089
4239	2012	1230
4585	2018	1259
50507	2038	1396
347735	2110	1407
1942	2200	1435
55147	2202	1536
2288	2213	1612
146057	2252	1780
23327	2328	1880
64943	2350	1896
8526	2662	2019
286467	2675	2038
5136	2790	2100
55089	2819	2123
414328	2822	2124
4801	2934	2212
201895	3075	2213
64359	3290	2239

7035	3554	2246
79776	3640	2359
115294	3745	2512
353141	3751	2533
5364	3910	2628
6721	4060	2662
84888	4190	2745
9839	4352	3045
605	4481	3055
9742	4828	3059
4287	4857	3108
1735	4883	3269
9910	4886	3310
1730	4889	3357
3985	5101	3570
23345	5207	3587
23433	5602	3663
23327	5724	3682
317762	5733	3684
11252	5781	3689
5859	5959	3702
27145	6445	3784
79760	6545	3920
1E+08	6913	3936
79664	7345	3937
23326	7360	4004
4651	7499	4094
54973	8034	4129
8869	8076	4190
92521	8322	4239
331	8814	4481
23598	8839	4688
7111	8992	4689
3276	9370	5027
27319	9459	5169
55005	9509	5184
81932	9926	5212
5701	9940	5294
8839	10008	5641
7157	10060	5737
5136	10110	5740

11252	10659	5742
6659	10810	5788
84105	11244	5800
10025	23433	6039
9547	23460	6275
5898	23531	6281
2196	23743	6335
7082	25890	6347
4287	25939	6348
51750	25975	6495
1856	26166	6503
6198	26353	6622
28987	26577	6629
6597	26872	6690
23200	27129	6850
158056	27286	6916
151258	29796	7096
92126	29958	7098
342035	29969	7111
53342	29995	7251
9508	50507	7409
345557	50862	7454
6474	50863	7462
54796	51421	7850
647121	51655	7942
259230	51716	8417
10476	51776	8540
22930	53342	8705
55714	54221	8843
780	55024	8876
1119	55286	9046
8349	55701	9051
51082	55819	9056
10457	56246	9182
55573	56603	9296
10523	56937	9308
1375	57124	9340
80323	57419	9473
4208	57623	9595
6159	58533	9863
27097	63924	9935

8876	63982	10216
286144	64081	10333
65124	64168	10476
7050	64219	10553
162394	64231	10638
79649	64400	10673
27250	64757	10904
644192	79190	10981
79191	79614	11010
10607	79689	11025
80143	79712	11026
2788	79987	11027
6990	80704	11309
5239	80760	11314
3609	80830	22797
5144	80854	22800
159013	81603	22918
122616	83445	23136
79869	83473	23484
8884	84674	23545
9654	84883	23547
8835	84886	23582
10673	84935	25798
2060	84981	27010
91612	85477	27128
160428	90135	27287
51119	116328	28974
9839	117581	29095
29894	117854	29760
84448	123879	29919
3714	126364	30009
8541	130399	50507
10479	130827	50856
27346	134145	51071
1829	137075	51167
4857	145376	51234
84265	151306	51255
84231	151651	51284
5552	151887	51338
5167	152302	51365
5136	154807	51371

6386	166336	51411
5883	196047	51635
6598	206338	51744
10491	254228	53353
404201	259217	54469
4886	285386	54491
285753	301	54504
284	338094	54952
1938	338382	54968
1182	339834	55013
5412	339983	55016
51547	340719	55052
6305	348094	55089
54873	388650	55203
27235	389033	55322
8720	389136	55328
64400	389756	55505
1E+08	400649	55799
727910	400707	55803
1154	414328	55808
51348	643783	55840
5239	644189	56000
84747	653140	57150
51084	801	57217
284217	1038	58484
6495	5376	63898
51490	6510	64092
55506	9604	64174
4094	10114	64333
8876	11237	64976
9444	26106	79145
51108	83890	79166
738	84215	79895
55799	1E+08	79953
7919	1E+08	80231
30812	122953	80342
514	169200	80762
140462	285386	80896
23644	388690	83464
57473	399951	83658
6624	402560	84079

22998	1	84437
388886	58	84448
6472	70	84519
5105	93	84868
644	222	85013
26578	273	85477
10919	321	89857
51477	384	90523
51338	534	91851
81706	575	93589
27316	607	114926
22833	634	115286
55819	711	117289
3075	770	117854
57186	784	122970
388815	883	128387
389058	923	130814
6526	1002	133418
4744	1082	147525
2662	1108	149351
586	1119	199221
55389	1154	199857
9240	1164	205251
8853	1181	219833
2108	1196	219855
28987	1287	220213
7037	1288	221143
80790	1294	260293
723790	1298	282969
6198	1299	283078
83857	1302	283316
375010	1382	283481
9976	1488	284013
10251	1592	284106
83648	1606	284593
4004	1644	284942
142678	1663	285440
8499	1746	339390
157773	1757	414308
4257	1775	414328
151254	1816	493861

201134	1829	644189
10399	1846	646278
5424	1911	653361
3005	1933	729515
283464	1937	
27250	1938	
1879	1942	
4651	2011	
25959	2091	
1663	2115	
6159	2134	
8289	2155	
9569	2187	
54751	2275	
121512	2288	
1499	2319	
23580	2521	
6660	2535	
8837	2593	
10743	2625	
8884	2686	
57332	2786	
9770	2788	
139818	2794	
130814	2859	
9945	2863	
8971	2877	
6335	2923	
25939	2962	
27237	3005	
51614	3081	
940	3150	
93589	3249	
10490	3276	
1E+08	3280	
131870	3321	
57188	3371	
57559	3609	
8600	3675	
116039	3714	
9869	3728	

9532	3732	
8772	3748	
9659	3755	
144363	3770	
10371	3778	
554203	3782	
3012	3816	
1181	3852	
10160	3902	
1213	3978	
50628	3980	
8825	4058	
84219	4143	
1E+08	4174	
22902	4192	
84288	4293	
130399	4355	
10371	4485	
9936	4486	
664	4488	
64420	4494	
91543	4744	
9968	4798	
129685	4884	
54221	5129	
7919	5137	
4926	5190	
5338	5296	
10919	5318	
4644	5324	
124583	5355	
6741	5413	
4907	5424	
645513	5427	
1240	5514	
4094	5524	
9820	5569	
64400	5596	
441094	5625	
1131	5634	
1511	5831	

10291	5883	
55831	5915	
80723	5916	
5130	5984	
23077	6097	
158295	6122	
22822	6136	
23002	6137	
285148	6139	
493869	6141	
55885	6142	
94240	6152	
63982	6158	
29887	6159	
30827	6167	
5141	6170	
317772	6189	
1193	6191	
115361	6208	
51209	6210	
3835	6228	
5066	6229	
116255	6242	
84719	6253	
23403	6307	
5834	6334	
5424	6421	
9136	6599	
256355	6636	
57404	6652	
2542	6714	
81029	6721	
80233	6764	
9757	6770	
9641	6926	
339344	6932	
8905	7015	
2662	7157	
730	7169	
5734	7249	
205327	7349	

38	7364	
5980	7365	
139065	7474	
151827	7551	
388815	7593	
969	7625	
4841	7637	
64794	7748	
5627	7753	
85377	7760	
7205	7775	
51278	7780	
1969	7916	
151556	7991	
6579	8019	
10395	8021	
54518	8086	
10061	8089	
327	8187	
54972	8189	
6414	8237	
2054	8241	
6139	8359	
80723	8481	
6445	8553	
79711	8570	
23654	8572	
8705	8632	
25849	8798	
830	8861	
6666	8906	
3075	8926	
6647	8927	
8484	9047	
81493	9061	
9473	9092	
647121	9136	
6941	9145	
23002	9235	
27316	9254	
1666	9394	

206338	9456	
57419	9466	
902	9468	
9353	9654	
23301	9742	
4883	9746	
9368	9820	
283078	9918	
80154	9925	
8228	10025	
29081	10083	
130026	10124	
729046	10140	
2690	10152	
57708	10155	
8073	10224	
23598	10256	
3344	10280	
5130	10385	
3908	10399	
339352	10522	
4762	10535	
8325	10610	
79974	10720	
4194	10740	
10157	10763	
27005	10765	
9353	10849	
2686	10868	
205327	10886	
22797	10902	
1E+08	10919	
9918	10951	
160897	10961	
83879	10998	
9098	11013	
50940	11039	
2002	11147	
4849	11180	
3269	11223	
353134	11237	

644538	11253	
9976	22838	
388403	22873	
2837	22883	
29887	22937	
55722	23081	
10097	23082	
81628	23089	
27344	23126	
5634	23205	
2581	23217	
10513	23269	
728142	23316	
54469	23363	
27023	23403	
55529	23421	
3067	23481	
191	23521	
5442	23523	
221895	23542	
55752	23563	
9686	23598	
552889	23640	
26099	23647	
7117	26000	
147015	26013	
125113	26047	
10742	26059	
5531	26151	
5627	26167	
4286	26233	
23028	26261	
285966	26354	
284441	27005	
3985	27092	
1E+08	27124	
55843	27316	
11031	27346	
197335	27440	
84056	29789	
38	30000	

7125	30845	
91526	50636	
4240	50861	
4644	51053	
2627	51116	
1577	51176	
7534	51477	
387758	51514	
28962	51559	
9444	51599	
91584	51750	
8992	53635	
340947	54094	
4094	54487	
55012	54512	
51170	54535	
1580	54549	
51277	54880	
54796	54972	
253935	54997	
388341	55072	
619426	55124	
1036	55144	
9590	55147	
122786	55190	
4060	55214	
58533	55311	
95	55374	
23654	55621	
23136	55657	
7099	55715	
354	55738	
8525	55765	
92906	55859	
353355	56033	
92521	56143	
65108	56256	
728131	56924	
767	56949	
79883	56974	
22921	57106	

824	57291	
551	57332	
79987	57497	
2206	57555	
151556	57613	
51185	57680	
51527	57695	
55374	57713	
780	57714	
152485	57820	
1787	57821	
1838	58500	
79987	60314	
6189	60681	
8774	64064	
10800	64073	
2078	64359	
965	64763	
440712	65057	
1E+08	65095	
57118	65108	
51115	65243	
1E+08	65266	
51660	65267	
4150	65989	
4627	79022	
6468	79025	
55352	79101	
122553	79140	
55757	79160	
1591	79690	
57134	79805	
1718	79861	
801	79869	
4889	79915	
2213	79957	
79137	79971	
55799	80032	
5908	80154	
11079	80162	
5166	80344	

2122	80347	
27352	80775	
3054	80863	
55380	81618	
6520	81831	
51024	81857	
10489	83650	
645	83743	
11107	83871	
7625	84057	
1E+08	84197	
7186	84221	
4326	84249	
10490	84271	
8906	84292	
3841	84303	
10226	84365	
9743	84433	
23179	84451	
6189	84461	
84891	84501	
54553	84532	
219749	84561	
29882	84717	
10351	84798	
23363	84856	
79739	84865	
7249	84889	
6134	84914	
10558	84922	
23085	84960	
1738	84970	
8322	84986	
1937	85028	
9590	85301	
375790	85320	
3675	90233	
284232	90506	
22871	90522	
388152	90525	
2905	90678	

79625	90701	
7755	91227	
63976	91522	
390705	91975	
23034	92292	
84498	92312	
1911	92714	
55333	112724	
57404	112885	
23208	112939	
5627	114044	
84293	114821	
57826	115509	
83544	115703	
6621	116840	
11154	120071	
6867	120224	
22822	122402	
646890	122616	
1984	123016	
216	124930	
2593	127281	
5689	127545	
221662	130612	
81571	134353	
843	136051	
6275	140578	
284185	140680	
51136	144203	
10439	144423	
4708	147341	
5689	147699	
284114	147968	
51115	148189	
1730	148252	
169792	150223	
116138	151230	
339390	158056	
55179	163154	
439951	163786	
6601	164592	

9315	171391	
7629	196403	
79611	196968	
134957	197258	
5279	200058	
5739	200172	
256355	200765	
339047	220433	
4254	221914	
118432	254887	
127943	256355	
53373	267002	
10097	283989	
79843	284086	
197258	284217	
56652	284244	
23492	284339	
54968	284889	
8817	284904	
6474	285986	
5950	286826	
55796	333926	
2823	339047	
23276	339479	
9632	339665	
23647	340286	
57097	349114	
6764	353149	
57130	360200	
5050	373863	
115265	375790	
2790	378825	
1852	388152	
3570	388610	
11213	388886	
84261	389127	
64097	389404	
6990	389435	
6159	390183	
55704	390595	
80856	390705	

123803	391081	
441430	391132	
1029	391777	
85477	392008	
147808	392522	
116447	400818	
1131	401105	
9736	402176	
808	441294	
84461	442075	
155435	548321	
10905	552889	
143187	643669	
1E+08	645159	
80267	645683	
19	647946	
23005	728411	
5101	728485	
79785	728732	
6508	729046	
170680	729279	
4154	731688	
83641	775	
9043	996	
7026	1196	
23208	5590	
8905	6170	
8082	6234	
5352	8237	
84271	8396	
91937	10521	
54928	11039	
130026	23114	
493869	23523	
644189	80052	
843	80154	
23643	80279	
5908	84133	
6400	84771	
63922	84950	
5567	91227	

7010	92312	
22875	1E+08	
64081	1E+08	
5140	1E+08	
9475	1E+08	
8943	1E+08	
163732	1E+08	
10522	1E+08	
284	1E+08	
136319	1E+08	
22919	121551	
140688	150244	
79175	150291	
64321	166994	
3603	253650	
2050	344423	
6909	344875	
6464	373861	
9618	387937	
2009	402562	
221806	402694	
116988	440030	
444	440703	
1666	445582	
55704		
29915		
3728		
114991		
23593		
6223		
55727		
26272		
1153		
83541		
375061		
683		
84552		
10878		
2819		
9194		
55139		

443		
2444		
6159		
122616		
79797		
79839		
635		
9050		
137964		
790		
5818		
8394		
55147		
7593		
90411		
161725		
50512		
57666		
6776		
1984		
7297		
51365		
3075		
4482		
10216		
Stroma	Epithelium	Immune
19	19	640
32	48	643
48	132	6354
51	133	1232
111	155	930
133	283	914
147	310	919
153	323	940
154	338	916
157	343	917
185	477	920
187	540	923
217	573	968
230	599	8832
284	644	925

364	664	10225
368	665	10563
398	666	6374
412	686	1536
443	727	79368
444	734	2335
477	830	50943
493	858	2358
551	1028	11251
554	1185	3004
635	1362	3112
650	1528	29851
654	1738	3560
762	1739	3568
781	1909	3577
794	1979	3579
847	2247	9834
857	2258	3902
858	2329	3932
859	2632	10288
869	2673	4094
943	2690	931
948	2729	4481
1012	2770	9437
1030	2790	4790
1031	2822	5133
1036	2908	29126
1050	2981	5473
1053	3032	5551
1066	3099	4068
1071	3225	55423
1160	3351	84868
1268	3419	608
1282	3679	23043
1284	3799	7185
1290	4023	9319
1306	4047	7535
1311	4122	
1368	4128	
1393	4139	
1464	4199	

1551	4223	
1580	4257	
1645	4259	
1728	4329	
1730	4352	
1776	4522	
1796	4547	
1842	4637	
1879	4901	
1892	4952	
1901	5091	
1962	5095	
2012	5105	
2034	5106	
2038	5151	
2042	5202	
2100	5207	
2110	5291	
2122	5352	
2171	5441	
2180	5525	
2184	5537	
2200	5602	
2273	5728	
2321	5824	
2632	5836	
2690	5864	
2701	5896	
2791	5950	
2819	6010	
2822	6272	
2828	6309	
2852	6342	
2878	6532	
2888	6573	
2977	6647	
2981	6660	
3002	7003	
3099	7068	
3142	7082	
3215	7098	

3216	7263	
3218	7301	
3240	7328	
3250	7442	
3290	7837	
3339	7855	
3384	8034	
3569	8544	
3620	8555	
3672	8674	
3679	8748	
3694	8760	
3745	8790	
3764	8800	
3791	8803	
3834	8825	
3903	8843	
3910	8908	
3921	8987	
3956	8997	
3991	9057	
3995	9194	
4017	9340	
4023	9411	
4091	9465	
4128	9475	
4162	9530	
4190	9588	
4199	9734	
4223	9749	
4232	9926	
4248	9936	
4257	9992	
4259	10046	
4311	10054	
4584	10079	
4826	10099	
4828	10239	
4855	10247	
4881	10390	
4883	10424	

4889	10558	
4897	10724	
4921	10810	
5091	10820	
5101	10840	
5105	10867	
5125	10974	
5138	11037	
5140	11099	
5153	11145	
5166	11163	
5168	11244	
5207	11260	
5209	11279	
5224	22800	
5236	22837	
5264	22871	
5320	22875	
5352	22908	
5443	22917	
5468	22921	
5502	23026	
5519	23032	
5577	23142	
5602	23180	
5732	23199	
5745	23235	
5787	23271	
5797	23285	
5802	23365	
5836	23433	
5837	23483	
5924	23518	
5950	23531	
5959	23554	
6285	23568	
6291	23593	
6319	23673	
6329	23766	
6445	23788	
6453	25798	

6492	25915	
6495	25966	
6507	26166	
6512	26190	
6517	26272	
6623	27067	
6660	27152	
6678	27178	
6711	28512	
6913	28962	
6939	29081	
7010	29092	
7060	29761	
7079	29887	
7108	50486	
7145	50649	
7293	50865	
7301	50999	
7345	51015	
7436	51029	
7532	51031	
7837	51074	
7855	51092	
7881	51099	
8034	51115	
8076	51175	
8322	51652	
8436	51761	
8483	53353	
8490	54414	
8547	54469	
8601	54494	
8622	54545	
8639	54566	
8644	54619	
8659	54675	
8671	54928	
8676	54996	
8694	55137	
8736	55193	
8829	55217	

8839	55335	
8843	55389	
8908	55449	
8987	55714	
9021	55742	
9034	55766	
9057	55777	
9194	55819	
9270	55825	
9340	55831	
9397	55876	
9459	55902	
9478	55973	
9507	56121	
9588	56900	
9628	56922	
9729	56995	
9734	57088	
9811	57496	
9899	57508	
10052	57515	
10082	57544	
10099	57546	
10110	57573	
10216	57600	
10219	58533	
10249	60370	
10316	63951	
10400	63971	
10449	64072	
10555	64081	
10580	64089	
10655	64121	
10810	64219	
10840	64395	
10965	64757	
10979	64940	
11067	64968	
11145	65084	
11217	79443	
11343	79611	

22795	79639	
22808	79739	
22821	79843	
22833	79863	
22837	79905	
22885	79959	
22899	80054	
22917	80318	
23026	80704	
23037	80705	
23057	80869	
23180	83452	
23187	83473	
23189	83737	
23235	83755	
23433	83935	
23452	84056	
23531	84071	
23630	84107	
23743	84108	
25924	84263	
25966	84265	
26011	84272	
26112	84293	
26166	84418	
26577	84542	
27129	84696	
27253	84883	
27286	84961	
28999	85027	
29887	85417	
29923	89910	
29944	90874	
29958	91137	
29969	91404	
29995	91584	
30846	91768	
50486	92689	
50507	92797	
50509	93587	
50863	112487	

50940	112703	
51085	112817	
51129	118429	
51226	119032	
51299	122773	
51308	122970	
51655	125875	
53353	126433	
54331	128387	
54438	128486	
54518	130399	
54674	131544	
54749	131870	
54873	132720	
54884	133383	
54988	133686	
55200	134145	
55273	134548	
55281	137872	
55283	140738	
55335	142891	
55714	142940	
55825	148534	
55885	148898	
55908	150709	
56246	150962	
56301	151742	
56603	151827	
56670	152503	
56901	152831	
56920	154807	
56937	157506	
56997	160140	
57101	160897	
57104	168448	
57477	196527	
57484	200558	
57514	205327	
57538	206338	
57546	219409	
57678	219736	

58160	220296	
60370	222166	
63876	254251	
63923	254778	
63924	254863	
64102	256302	
64399	257397	
64641	267004	
64714	283135	
64757	283481	
78989	284366	
79148	285148	
79190	285343	
79611	286205	
79614	317649	
79686	339983	
79689	340351	
79746	349152	
79827	374354	
79905	374986	
79974	375449	
80054	388403	
80235	388650	
80243	399474	
80704	399512	
80723	400604	
80760	401264	
80830	439938	
81544	440503	
81575	494143	
81792	572558	
83401	641654	
83595	644189	
83636	644242	
83643	645513	
83660	730102	
83733	768211	
83878	1E+08	
83888	1E+08	
84230	1E+08	
84263	1E+08	

84293	59	
84570	72	
84649	191	
84688	240	
84701	244	
84706	271	
84883	330	
84981	362	
85027	415	
85329	478	
85411	480	
85477	481	
90135	525	
90523	639	
90586	752	
90952	816	
91461	864	
91543	914	
91584	917	
91851	923	
92014	939	
92162	962	
94233	973	
112703	1107	
112817	1117	
114036	1139	
114780	1164	
114804	1236	
115330	1294	
115361	1381	
115827	1400	
116255	1455	
116328	1524	
116362	1525	
117248	1535	
125875	1606	
126433	1616	
126567	1729	
130399	1731	
134265	1838	
136853	1915	

139818	1933	
140609	1937	
140706	2049	
140738	2120	
148534	2185	
148898	2275	
151306	2352	
151827	2528	
152302	2568	
152573	2919	
152831	3007	
153579	3009	
154807	3068	
157506	3291	
161176	3493	
163782	3500	
168667	3507	
170626	3512	
196527	3514	
201562	3575	
203260	3669	
206338	3710	
220296	3779	
221883	3782	
222166	3783	
222962	3815	
253635	3860	
253738	3866	
254228	3883	
255877	3898	
256435	3932	
259217	3957	
283481	3984	
286753	3985	
338094	4050	
338442	4057	
339834	4065	
339983	4072	
342035	4744	
348093	4794	
359845	4804	

374387	4908	
375061	5026	
389756	5150	
392636	5165	
401052	5284	
401491	5368	
440503	5450	
641371	5588	
641654	5590	
644242	5596	
644662	5883	
646278	6125	
646962	6139	
653140	6141	
727910	6175	
729359	6187	
1E+08	6188	
1E+08	6189	
9	6193	
12	6195	
72	6201	
127	6208	
136	6223	
214	6233	
220	6242	
222	6374	
245	6398	
247	6447	
270	6523	
288	6541	
290	6590	
360	6663	
362	6690	
374	6932	
399	7059	
481	7097	
525	7136	
563	7277	
608	7421	
638	7462	
688	7748	

721	7791	
722	7852	
758	8120	
760	8481	
771	8508	
780	8631	
816	8645	
827	8787	
866	8906	
928	9252	
999	9304	
1001	9394	
1040	9595	
1116	9654	
1117	9658	
1264	9684	
1280	9744	
1287	9762	
1288	10045	
1294	10123	
1296	10158	
1298	10293	
1308	10319	
1356	10331	
1364	10360	
1365	10399	
1366	10460	
1515	10514	
1524	10633	
1525	10681	
1573	10801	
1592	10850	
1594	11040	
1641	11151	
1741	11184	
1832	11211	
1875	11240	
1907	11262	
1945	22823	
1999	22889	
2001	23127	

2049	23195	
2064	23223	
2065	23225	
2066	23550	
2134	23650	
2151	25791	
2161	25797	
2274	25884	
2330	25894	
2348	26140	
2527	26232	
2568	26298	
2625	26579	
2678	26586	
2679	27040	
2886	27065	
2925	27071	
2939	27436	
3084	28299	
3169	28461	
3249	28778	
3291	28831	
3294	28923	
3493	29122	
3512	29842	
3664	29909	
3673	29997	
3696	30817	
3775	30845	
3783	50617	
3800	50619	
3815	50802	
3852	50805	
3854	50852	
3855	50861	
3860	51090	
3861	51237	
3866	51303	
3868	51316	
3872	51435	
3875	51497	

3882	51537	
3883	51659	
3887	51676	
3892	51700	
3898	54097	
3963	54332	
4038	54361	
4057	54753	
4065	54763	
4070	54855	
4072	54881	
4151	55073	
4192	55692	
4316	55964	
4435	56606	
4488	56652	
4582	57035	
4602	57106	
4645	57111	
4680	57291	
4920	57348	
4935	57555	
4950	57561	
5017	57699	
5021	57823	
5029	64110	
5174	64170	
5241	64359	
5266	64753	
5268	64780	
5284	64926	
5318	65268	
5327	65987	
5349	79077	
5357	79098	
5450	79370	
5521	79674	
5570	79703	
5590	79754	
5596	79888	
5603	80005	

5618	80045	
5629	80237	
5649	80342	
5652	81557	
5655	81628	
5744	83450	
5753	83593	
5764	83858	
5803	83879	
5806	84057	
5874	84059	
5918	84218	
5932	84679	
5947	84707	
5997	84766	
6097	84876	
6296	84941	
6297	84952	
6337	84958	
6366	85409	
6385	90632	
6447	90701	
6523	91319	
6558	91353	
6583	92196	
6590	92304	
6653	92312	
6662	93082	
6663	115650	
6690	120425	
6692	121504	
6820	121642	
6833	122589	
6898	126147	
6947	129080	
7020	131177	
7021	136306	
7022	145864	
7025	146439	
7033	147138	
7042	147798	

7113	148641	
7136	150365	
7162	152015	
7163	154043	
7227	164284	
7364	171177	
7365	200298	
7366	200634	
7368	201176	
7421	220717	
7447	221002	
7465	221178	
7474	221188	
7494	253650	
7504	256355	
7718	283234	
7802	283551	
7976	333929	
8001	337879	
8045	337968	
8120	374403	
8190	376693	
8324	388115	
8416	389634	
8437	391777	
8537	439949	
8572	440180	
8612	474170	
8614	548596	
8626	643779	
8711	645367	
8787	652070	
8842	654434	
8913	728411	
9076	728640	
9077	729046	
9256	1E+08	
9413	1E+08	
9455	1E+08	
9456	1E+08	
9497	1E+08	

9618	1E+08	
9620	1E+08	
9633	1E+08	
9651	1E+08	
9687		
9914		
9915		
9957		
9982		
10002		
10045		
10053		
10098		
10103		
10158		
10207		
10256		
10265		
10267		
10279		
10309		
10319		
10331		
10451		
10529		
10537		
10538		
10551		
10570		
10633		
10647		
10648		
10653		
10720		
10848		
11012		
11013		
11122		
11187		
11202		
11226		

11240		
11341		
22874		
22941		
22996		
23086		
23089		
23242		
23286		
23336		
23423		
23541		
23550		
23650		
23767		
25800		
25803		
25884		
25984		
26022		
26232		
26256		
26289		
26298		
26504		
27076		
27087		
27122		
27134		
27190		
27232		
27285		
27293		
27295		
28299		
28461		
28831		
28965		
29122		
29841		
29842		

50617		
50805		
51361		
51806		
53335		
54033		
54097		
54101		
54361		
54762		
54763		
54836		
54845		
54855		
54869		
54894		
55040		
55061		
55107		
55227		
55349		
55359		
55503		
55512		
55521		
55540		
55612		
55620		
55655		
56171		
56477		
56521		
56675		
56924		
57111		
57221		
57402		
57530		
57535		
57549		
57593		

57596		
57604		
57619		
57648		
57662		
57822		
58495		
59084		
59277		
59352		
63947		
64063		
64065		
64284		
64388		
64699		
64787		
64866		
65055		
65266		
65268		
65983		
65987		
78986		
79056		
79083		
79092		
79098		
79152		
79170		
79191		
79192		
79589		
79608		
79642		
79669		
79674		
79679		
79703		
79730		
79767		

79781		
79838		
79841		
79875		
79948		
79977		
80004		
80131		
80144		
80154		
80162		
80303		
80336		
80736		
81539		
81557		
81706		
81831		
81849		
83450		
83481		
83690		
83959		
83987		
84033		
84059		
84069		
84125		
84189		
84419		
84440		
84553		
84612		
84679		
84707		
84709		
84866		
84951		
84952		
84953		
84958		

84966		
85320		
85415		
89765		
90019		
90161		
90293		
90835		
91227		
91319		
91353		
91409		
91683		
91862		
91894		
92196		
92291		
92292		
92304		
92359		
92421		
92565		
93082		
93099		
93517		
94009		
112399		
114907		
115572		
116844		
118430		
118932		
119391		
120071		
120224		
121506		
123036		
124056		
124739		
124975		
126006		

126147		
126695		
126969		
127602		
127733		
127845		
128218		
130574		
130576		
131177		
138065		
139411		
140597		
144347		
144501		
145757		
146330		
146439		
147798		
147968		
148327		
148641		
149428		
149461		
149563		
150696		
152015		
153478		
153562		
155465		
157574		
157869		
158584		
161497		
163183		
163702		
170685		
171177		
199964		
200634		
200879		

202333		
203328		
219623		
219970		
220963		
221002		
221806		
222865		
253190		
254887		
255743		
260436		
283177		
283454		
284085		
284252		
284348		
284654		
284656		
286077		
286887		
317754		
333926		
337968		
338440		
339010		
340542		
342667		
346389		
346689		
347735		
349149		
376267		
377677		
387914		
388115		
388610		
389336		
389432		
400451		
400573		

400618		
401237		
401474		
404785		
440224		
440335		
441376		
442249		
445328		
548596		
553158		
642273		
644945		
645367		
646090		
652070		
653499		
728441		
1E+08		
1E+08		
1E+08		
1E+08		
1E+08		
1E+08		
1E+08		
P53	Hypoxia	Proliferation
9520	133	332
83464	51129	891
595	54541	991
2625	2171	83540
345757	55640	55165
51313	51083	10403
56204	10397	4288
123036	5351	9232
85004	4860	6241
7844	58528	7298
9338	9123	11065
4756	7345	
2540936	7422	
23048		
9674		

6416		
29028		
7832		
1163		
983		
994		
995		
1063		
55165		
54069		
890		
1033		
1643		
2305		
8836		
29781		
9493		
4085		
4172		
4756		
4521		
9232		
5424		
5550		
5984		
6790		
25800		
7153		
6890		
7107		
1026		
3833		
92421		
9319		
55536		
7277		
11065		
4605		
